# Grade 2 disabilities in leprosy patients from Brazil: Need for follow-up after completion of multidrug therapy

**DOI:** 10.1371/journal.pntd.0006645

**Published:** 2018-07-16

**Authors:** Marcos Túlio Raposo, Martha Cerqueira Reis, Ana Virgínia de Queiroz Caminha, Jörg Heukelbach, Lucy Anne Parker, Maria Pastor-Valero, Maria Ines Battistella Nemes

**Affiliations:** 1 Departamento de Saúde I, Universidade Estadual do Sudoeste da Bahia, Jequié, Bahia, Brazil; 2 Instituto de Saúde Coletiva, Universidade Federal da Bahia, Salvador, Bahia, Brazil; 3 Departamento de Saúde Comunitária, Universidade Federal do Ceará, Fortaleza, Ceará, Brazil; 4 Departamento de Salud Pública, Historia de la Ciencia y Ginecología, Universidad Miguel Hernández, Alicante, Alicante, Spain; 5 CIBER Epidemiología y Salud Pública (CIBERESP), Madrid, Spain; 6 Departamento de Medicina Preventiva, Universidade de São Paulo, São Paulo, São Paulo, Brazil; Swiss Tropical and Public Health Institute, SWITZERLAND

## Abstract

**Background:**

Leprosy continues to be a public health problem in many countries. Difficulties faced by health services include late diagnosis, under-reporting of new cases, adequate monitoring of disabilities and treatment. Furthermore, systematic follow-up after completion of treatment is important, when new disabilities may occur, or existing disabilities may get worse. The objective of the present study was to determine the prevalence of leprosy-associated grade 2 disabilities (G2D) after completion of multidrug therapy (MDT) and to identify factors associated with G2D.

**Methods:**

We performed a cross-sectional study of 222 leprosy cases registered in Vitória da Conquista, Bahia state, Brazil from 2001–2014. We performed a clinical examination of the study participants and collected socio-economic and clinical information by interview. We identified factors associated with grade 2 disability (G2D) using logis tic regression.

**Results:**

In total, 38 (17.1%) participants were diagnosed with G2D, and 106 (47.7%) with grade 1 disabilities (G1D). The following independent factors were significantly associated with G2D: occurrence of leprosy reaction (adjusted OR = 2.5; 95%CI = 1.09–5.77), thickening and/or tenderness of one or more nerve trunks (adjusted OR = 3.0; CI = 1.13–8.01) and unemployment (adjusted OR = 7.17; CI = 2.44–21.07).

**Conclusions:**

This study shows that physical disabilities remain after completion of MDT and frequently occur in an endemic area in Brazil. Finding new ways to reduce the burden of disability are urgently needed, and may include systematic follow-up of patients after treatment completion combined with evidence-based preventative measures.

## Author summary

Leprosy is a Neglected Tropical Disease that is still common in many countries. Patients who have had leprosy often suffer long-term physical disabilities. For some patients, disabilities occur after finishing treatment or existing disabilities may get worse. We contacted 222 leprosy patients who had previously completed multidrug therapy (MDT) in a typical endemic area in Brazil and assessed leprosy-associated disabilities, and the associated clinical or sociodemographic factors. We found that 17.1% of the participants had severe (grade 2) disabilities. Disabilities were more common among patients who had had a leprosy reaction, thickening and/or tenderness of one or more nerve trunks and were unemployed. The findings are important because we show that physical disabilities occur frequently after treatment completion in this area, and that patients need long-term follow-up by the health system. Integrated measures that consider clinical and socio-economic aspects are also needed to reduce leprosy burden after completion of treatment, such as devising of public policies, implementation of clinical monitoring and the development of evidence-based guidelines for follow-up after release from MDT.

## Introduction

In 2016, a total of 214,783 leprosy cases were reported globally (case detection rate: 2.9/100,000 population), of which 11.7% (25,218) occured in Brazil [1] (case detection rate: 12.2/100,000 population) [2]. Within the country, the prevalence is highest in the poorer regions of the North, Mid-West and Northeast [3]. The case detection rate in the Northern state of Bahia was 13.6/100,000 population, with a rate of 15/100,000 population in the city of Vitória da Conquista, located in the Southeastern part of the State [2]. Concerning G2D, globally, the rate of new G2D cases was 1.7/million population [1]. The G2D rate of 8.4/million population was registered in Brazil, 6.2/million population in Bahia and in Vitória da Conquista this rate was 17.3/million population, twice the national average [2].

Leprosy may lead to considerable irreversible physical disabilities [4], exacerbated by poor socioeconomic conditions, stigma and late diagnosis [5]. The nerve damage caused by the disease can be sensory, motor and/or autonomic and may occur before diagnosis, during multidrug therapy (MDT) or after completion of treatment [6–9]. So-called leprosy reactions are the main cause of nerve damage and disabilities [10–12]. Early diagnosis and prompt treatment of these conditions can help to prevent the development or worsening of disabilities [13].

Overall detection rates have declined following the adoption of MDT as the standard treatment in 1991. However, the proportion of grade 2 disabilities (G2D) increased from 2006 to 2015, as a result of delayed diagnosis and possibly due to improvements in the quality of grade of disability reporting [14, 15]. In 2016, the proportion of visible deformities or Grade 2 Disability (G2D) among new cases, was 5.8% globally and 6.9% in Brazil. Although a reduction in G2D cases was registered in 2016, most likely as a result of community-based campaigns [1], the projected global G2D burden for 2020 is estimated at 1 million [16]. Grade of disability is a key epidemiological and operational indicator, where G2D is considered an indicator for delayed diagnosis and a hidden endemic [17].

The operational difficulties faced by primary health services are not limited to under-diagnosis and under-reporting of new leprosy cases. Monitoring of clinical progression, disabilities and treatment is challenging, and the lack of active follow-up after treatment completion is common. New disabilities or worsening of existing disabilities [18, 19], particularly due to leprosy reactions, can occur up to eight years and longer after treatment completion [20].

In this cross-sectional study, we reviewed 222 leprosy patients who had completed MDT in an endemic area in Brazil to identify the current proportion of physical disability and associated factors. The results will help shed light on the burden of disability among leprosy patients in this hyperendemic region, and will be useful to inform the design of effective control measures.

## Methods

### Ethics statement

Study procedures complied with the Declaration of Helsinki and the Research Ethics Committee of the Federal University of Ceará–Brasil reviewed and approved the study protocol (CAAE 19258214.2.000.5054). The Secretariat for Health of the State of Bahia and by the Municipal Secretariat of Health of Vitória da Conquista, granted permission to conduct the study. All study participants were over 18 years old of age and provided written informed consent. We referred all patients who required the attention of the healthcare services to the municipal health services, where they were received free assistance by specialists.

### Study area and population

A cross-sectional study was carried out in Vitória da Conquista, Bahia state, Brazil, a municipality with a population of 346,069 [21]. The study population comprised all new leprosy cases in the city registered by the National Disease Notification System (SINAN) and diagnosed between 01/01/2001 and 31/12/2014, regardless of time from releasing MDT, that had completed therapy at least one month prior to the study. Exclusion criteria included the following: incorrect address, change of address to outside the study area, death, refusal to participate in the study, failure to attend scheduled evaluations, language difficulties and cognitive deficits that impeded the understanding of the study.

### Data collection

We identified leprosy patients diagnosed during the study period using SINAN, a computer-based system collecting demographic and clinical data. Individuals were visited at their homes by a Community Health Worker (health professionals from the community involved in health promotion and prevention in primary care), and asked whether they would be willing to participate in the study. Subsequently, the health worker introduced the project team members to the potential participants. Study participants provided written informed consent. We collected complementary data on operational classification of leprosy, presence of leprosy reactions (at any given point prior to diagnosis and/or during MDT and/or after MDT completion), nerve thickening and/or tenderness, comorbidities, among others from clinical records, and cited the participants at the primary health clinics nearest their homes for physical examination and a structured questionnaire to collect socioeconomic and demographic data. Trained physiotherapists performed all physical examinations and were monitored by experienced researchers. We assessed sensory and motor functions of the eyes, hands and feet according to national guidelines (S1 Appendix) [13] to determine grade of disability. Briefly, grade 0 disability (G0D) represented normal sensation, no visible impairments; grade 1 disability (G1D) represented impaired sensation, but no visible impairments; and grade 2 disability (G2D) represented visible impairments/deformities and/or severe visual impairment (vision worse than 6/60; inability to count fingers at 6 metres) [22]. We also applied the “Screening of Activity Limitation and Safety Awareness” (SALSA) Scale, which assesses components of functioning and grades activity limitation and risk awareness based on an increasing scale from absence of limitation (10–24 points) to extreme limitation (60–80 points) [23].

### Variables and statistical analysis

We calculated the proportion of G2D, together with 95% confidence intervals. The presence or absence of G2D was the dependent variable in the statistical analysis. We defined G2D as visible disability detected at the clinical examination performed by the study team. The independent variables were distributed in two groups: (1) sociodemographic (age at study recruitment, gender, time since diagnosis, education, area of residence, per capita household income, employment status, participation in Brazil’s Family Allowance Program “Bolsa de Família”, drinking water supply, sanitation, waste collection, number of individuals per bedroom); and (2) clinical characteristics (operational classification, occurrence of leprosy reaction, nerve thickening and/or tenderness on palpation, time from diagnosis to study, comorbidities and activity limitation). Activity limitation was categorized as a binary variable based on the SALSA scale, whereby scores ≤ 24 indicated no limitation, and scores > 25 activity limitation [23].

We used Pearson chi-squared test to compare proportions of categorical variables. Odds Ratios (OR) were calculated to determine the association between G2D and the independent variables using age- and sex-adjusted logistic regression (LR) models. We used multivariable logistic regression using backward and forward stepwise procedures to build the models considering all variables associated with G2D in the crude analysis with a *P* values > 0.10. Covariates that changed original estimated coefficients (OR) by more than 20% were included as confounders. We excluded observations with missing values, to avoid spurious results [24]. We used the stepwise regression method to fit the multivariate logistic regression models. We used both backward elimination of variables and forward selection of variables. The significance of all variables was examined using a Wald test. If one or more variables were associated with *P* values > 0.10, the variable that had the highest *P* value was dropped from the model. The model was estimated again and the significance of the remaining variables reexamined using the same method as above. This iterative procedure continued until only variables with a significance level of at least 0.10 remained [25]. Statistical analysis was performed using Stata 14.0 (Stata Corporation, College Station, USA) [26].

## Results

**Fig 1:**
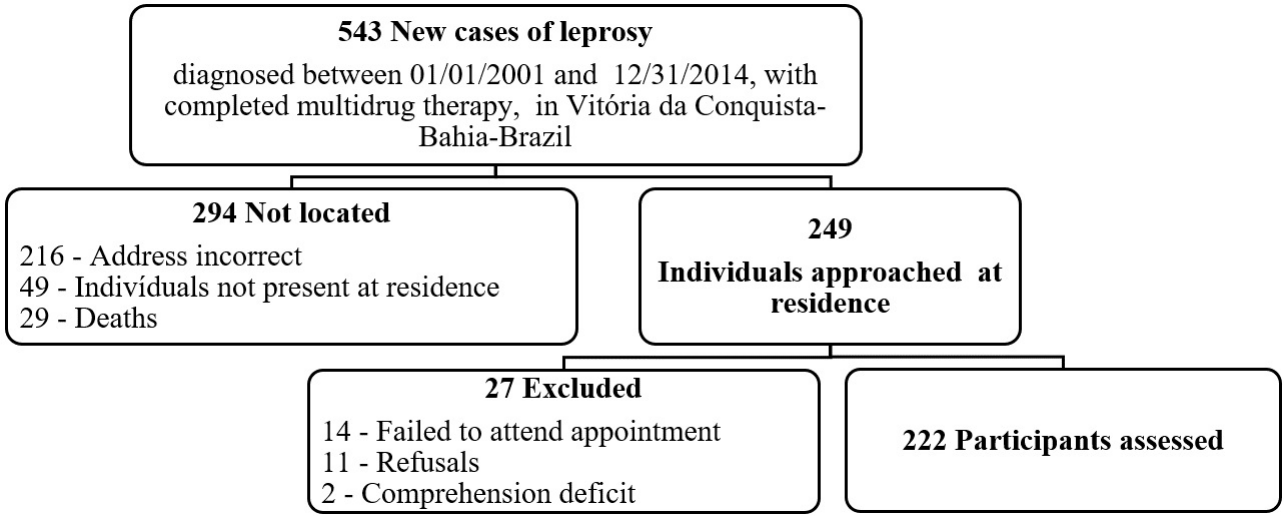
Selection process for leprosy cases with completed multidrug therapy, diagnosed between 2001 and 2014 in Vitória da Conquista, Bahia, Brazil.

There were 543 new leprosy cases diagnosed between 01/01/2001 and 31/12/2014 in Vitória da Conquista, Bahia state, Brazil who were eligible for inclusion in the study. Of these, 249 (45.8%) were located and invited to take part in the study. 14 were excluded for not attending the examination on the scheduled dates, 11 of whom refused to take part in the study, while 2 were excluded for neurological diseases affecting cognition and consequent collaboration during the clinical interview and physical examination. Of the individuals not located, 216 no longer resided at the address registered in SINAN, 49 were not found after three visits to the address registered in SINAN and 29 had died. Consequently, a total of 222 (40.9%) individuals were included in the study (Fig 1).

At the time of leprosy diagnosis, 106 (47.7%) individuals had been assessed for disability with 21.7% (95%CI: 14.8–30.7) classified as G1D and 19.8% (95%CI: 13.2–28.6) as G2D. At completion of MDT, disability grading was performed in only 77 (34.8%) individuals: 22.1% (95%CI: 14–32.9) G1D and 14.3% (95%CI: 7.9–24.2) G2D. At study recruitment, of the 222 participants, 38 (17.1%; 95% CI: 12.7–22.7) were diagnosed with G2D, and 106 (47.7%; 95%CI: 41.2–54.3%) with G1D. Only 46 individuals had the disability grading assessed at the three different time points (at diagnosis, at completion of treatment and at study recruitment). Among 46 individuals, 28 participants had had G0D at diagnosis, 12 (42.8%; 95%CI: 25.2–62.5%) of them had developed G1D and 1 (3.6%; 95%CI: 4.4–23.7%) had evolved to G2D in the period until they were recruited to the study.

Concerning age at diagnosis, there was a significant difference between mean age of individuals currently classified as G2D (47.1 ± 16.7 years) and those without G2D (42.9 ± 18.9 years) (p<0.008). At study recruitment the mean age of patients with G2D (60.7 ± 17 years) was higher than the mean age (53.2 ± 16.2 years) of those without G2D (p<0.010). The mean time from date of diagnosis to study inclusion was 7.48 (±3.5) years for the G2D group and this was not significantly higher than for those without G2D (p = 0.643) (Table 1). One in four of the participants in the lowest educational status (0–3 years) were diagnosed with G2D which was higher than the other educational groups although not statistically significant in all groups. Individuals with G2D more frequently unemployed, and G2D was associated with MB disease, leprosy reactions, nerve thickening and/or pain on palpation. Furthermore, they were classified more commonly with activity limitation (Table 1).

**Table 1:** Bivariate analysis of factors associated with grade 2 disability (G2D) in leprosy patients who had completed treatment in Vitória da Conquista, Bahia, Brazil (n = 222).

VARIABLES	Examined n^1^	G2D n (%)	OR (95% CI)	*P*-value
**SOCIODEMOGRAPHIC**				
**Gender**				
Male	114	23 (20.2)	1.57 (0.77–3.19)	0.216
Female	108	15 (13.9)	Reference	
**Education (years)**				
≥12	31	3 (9.7)	0.31 (0.09–1.12)	0.074
8–11	28	3 (10.7)	0.35 (0.10–1.26)	0.109
4–7	67	8 (11.9)	0.40 (0.17–0.95)	0.037
0–3	94	24 (25.5)	Reference	
**Area of residence**				
Rural	68	13 (19.1)	1.22 (0.58–2.57)	0.600
Urban	148	24 (16.2)	Reference	
**Per capita household income (minimum wage)**^2^				
< 45	94	17 (18.1)	1 (0.48–2.10)	1
≥ 0.45	94	17 (18.1)	Reference	
**Employment status**				
Inactive/Unemployed	117	33 (28.2)	7.70 (2.88–20.6)	<0.001
Active/Employed	103	5 (4.9)	Reference	
**“Bolsa Família Program”**^3^				
Yes	54	9 (16.7)	1.06 (0.47–2.40)	0.892
No	166	29 (17.5)	Reference	
**Safe drinking water**				
No	27	5 (18.5)	1.12 (0.39–3.16)	0.838
Yes	189	32 (16.9)	Reference	
**Sanitation**				
No	80	15 (18.8)	1.20 (0.58–2.47)	0.628
Yes	136	22 (16.2)	Reference	
**Waste collection**				
No	42	5 (11.9)	0.60 (0.22–1.65)	0.321
Yes	174	32 (18.4)	Reference	
**Individuals per bedroom**				
≥ 3	36	7 (19.4)	1.20 (0.48–3.01)	0.687
1–2	180	30 (16.7)	Reference	
**DISEASE–RELATED**				
**Operational classification**				
Multibacillary	140	29 (20.7)	2.38 (1.03–5.50)	0.042
Paucibacillary	81	8 (9.9)	Reference	
**Leprosy reaction**				
Yes	120	27 (22.5)	2.40 (1.13–5.13)	0.024
No	102	11 (10.8)	Reference	
**Nerve thickening and/or tenderness on palpation**				
Yes	148	32 (21.6)	3.13 (1.24–7.86)	0.015
No	74	6 (8.1)	Reference	
**Time from diagnosis to study exam (years)**				
≥7	115	21 (18.3)	1.20 (0.59–2.45)	0.615
<7	102	16 (15.7)	Reference	
**Chronic comorbity**^4^				
Yes	107	14 (13.1)	0.56 (0.27–1.16)	0.120
No	114	24 (21.1)	Reference	
**Activity limitation**^5^				
Yes	101	29 (28.7)	4.98 (2.15–11.54)	<0.001
No	107	8 (7.5)		

OR: Odds ratio; 95% CI: 95% Confidence interval.

^1^ Data not available for all individuals.

^2^ Estimated minimum wage for Brazil, in US$, at time of study: 1 minimum wage = US$ 261.00.

^3^ The “Bolsa Família Program” is a program of direct transfer of income that benefits households in poverty and extreme poverty in Brazil.

^4^ Self-reported high blood pressure and/or diabetes and/or hypercholesterolemia.

^5^ No = SALSA score (10–24); Yes = SALSA score (25–80).

The occurrence of leprosy reactions, presence of nerve thickening and/or pain on palpation, and unemployment were all independently associated with G2D (Table 2).

**Table 2:** Multivariate logistic regression analysis of factors associated with G2D, adjusted for sex and age, in Vitória da Conquista, Bahia, Brazil.

VARIABLE	Final Model^1^	
	Adjusted OR (95% CI)	*P*-value
Leprosy reactions	2.51 (1.09–5.77)	0.031
Nerve thickening and/or pain on palpation	3.01 (1.13–8.01)	0.028
Employment status (unemployed)	7.17 (2.44–21.07)	<0.001
Gender (male)	1.85 (0.83–4.08)	0.130
Age	1.01 (0.98–1.04)	0.447

OR: Odds ratio; 95% CI: 95% Confidence interval.

^1^ Variables in the final model: leprosy reaction, employment status, nerve thickening and/or pain on palpation, gender and age.

## Discussion

This study revealed a high disability burden among leprosy patients after completion of MDT in an endemic area in Brazil. The findings call for systematic and longitudinal care for individuals after treatment completion. We identified clinical and socio-economic factors associated with G2D that can be useful for health workers to plan and implement improved prevention and control measures.

The prevalence of G2D among patients who had completed MDT was 17.1%, which is higher than many previous reports, although within the 8.6–33.8% range reported [19, 27–31]. Potential explanations are that the average time since diagnosis was higher in our study than in others [20, 28] although the majority of studies did not report the time of diagnosis in them [19, 27, 29–31]. This finding highlights the need for active surveillance and continuity of long-term integrated care to reduce the impact of disabilities [17].

The analysis of sociodemographic variables showed, similar to other studies from India and Brazil [7, 30, 32], that G2D was associated with age, and that there is a high burden among groups of productive age [19, 27, 31, 33]. In our study, the unemployment rate among the population with G2D was high. Unemployment was associated with education and the other socioeconomic or environmental determinants analyzed. In addition to being a risk factor for G2D, it is likely that G2D itself is a triggering factor for unemployment and thus exacerbates the chain of unemployment and social vulnerability. However, other studies carried out in Brazil failed to find this association, perhaps due to differences in the socioeconomic level of the study participants [18, 27]. In line with previous studies [19, 34], the severe disability evidenced by G2D was associated with lower education, suggesting that formal education favors knowledge held by patients on healthcare needs, seeking of medical assistance and hence, greater access to health services [34, 35]. These factors associated with G2D have a socioeconomic impact, exacerbating the vicious cycle of poverty and inequalities and increasing the risk of deterioration of disabilities [9].

In this context, considering that socioeconomic status is associated with leprosy [36, 37], Brazil has adopted the strategy of decentralizing disease control measures to primary health care. In parallel, implementation of horizontal government programs to tackle extreme poverty, such as the “Family Allowance Programme–Bolsa Família”, have helped reduce the overall leprosy incidence on a national level [3]. Nevertheless, leprosy remains an important public health problem in Brazil, and the country is considered as highly endemic [1]. In the present study, we did not observe a significant association between participation in the ´Family Allowance Programme´ and G2D, perhaps owing to the overall low socioeconomic level of the residents in the study area.

The presence of non-communicable chronic comorbidities was not associated with G2D [27] in our study. This finding should not preclude joint actions of the leprosy control program in collaboration with other public health programs for chronic diseases, given their high prevalence in the general population and that treatment of comorbidities helps prevent disabilities [38].

Our findings are consistent with the literature indicating greater risk of nerve damage and, consequently, of permanent disabilities (G1D and G2D) in MB cases [6, 7, 9–11, 14, 27, 29, 34, 39]. We confirmed the association between MB clinical forms and the occurrence of visible deformities after completion of MDT [19, 28]. However, the lower occurrence of G2D in the paucibacillary group does not diminish the severity of the impairment, nor justify taking this group from the focus of priority actions.

In this study, the severity of reactions was in line with the recognized association of nerve damage and disabilities [10, 12, 17, 40]. Leprosy reactions, on a global level, exhibit high variability, and therefore our data are consistent with those of other studies, whose rates range from 20 to 57% [38, 41–46] and confirm the association between G2D and the occurrence of leprosy reaction at some point (prior to diagnosis, during MDT or after MDT completion) in the patient´s life [18, 19, 40].

The association of thickening and/or pain on palpation with G2D was also confirmed in previous studies [7, 34, 41], highlighting the need for continued neurological assessment after completion of the treatment regimen to prevent the onset and progression of disabilities in individuals exposed to the risk of disabilities and their determinants.

Given the high incidence of leprosy-related complications after MDT and the risk factors involved [19, 20, 27–31, 46, 47], lack of follow-up may lead to the need for further healthcare actions to manage potentially preventable complications [9, 19, 28]. The situation is particularly complicated after treatment completion because these patients are no longer monitored by public health services [9] and thus go largely unrecognized by the health authorities [20]. This study revealed a high prevalence of G2D patients with significant functioning problems after treatment. This patient group requires greater visibility in the political arena and integrated care should be provided [7, 48]. This care involves systematic monitoring of physical disabilities and tertiary rehabilitation, which includes a sustainable long-term approach to physical, psychological and social aspects [9, 19, 27, 28, 49].

The identified associated factors with G2D indicate the vulnerability affecting this group. The challenges providing integrated care for people affected by leprosy are increased, even after bacteriological cure, if not subjected to systematic follow-up and monitoring.

From the wider perspective of care for leprosy and people with disabilities, in line with the WHO Global disability action plan 2014–2021 and the Global Leprosy Strategy 2016–2020 [50, 51], this study renews discussion on the adoption of health policies promoting integrated care after MDT completion. The situation calls for the implementation of multi-sectoral policies and strategies to continue health surveillance after completion of MDT. Integrated control measures should include the development of ethics protocols, regulation of a system for reporting events post discharge, implementation of guidelines centered on the detection and timely treatment of disabilities and complications (such as leprosy reactions), monitoring factors associated with disabilities, and also the provision of interventions promoting self-care, disability prevention and availability of rehabilitation services.

Due to the retrospective nature of the study, we were unable to include all patients diagnosed with between 01/01/2001 and 31/12/2014 in Vitória da Conquista, Bahia state, Brazil. It is difficult to establish how the low participation rate has influenced the study results. On the one hand, it is possible that the patients underrepresented here are more likely to be marginalised populations of low socioeconomic position which have shown to be excluded from research in numerous studies. Given the strong relationship between social vulnerability and leprosy [32] we may be underestimating the true rates of G2D in this population. On the other hand, it is important to acknowledge that patients with severe disability caused by leprosy are more likely to be in contact with the health service and therefore more likely to have up-to-date medical records and be easier to trace. In this case, we will overestimate G2D. Either way, this does not detract from the findings in that a significant proportion of patients remain with significant disability after treatment completion. The study could therefore underestimate the true burden of disability among leprosy patients in this area. As we performed a cross-sectional study in a population that had completed MDT some time ago, causal relationships were difficult to assess, and data on associations should be interpreted with care. Information was missing in some cases, particularly regarding assessment of disabilities at diagnosis, during treatment and upon MDT completion. Social variables such as employment were only collected at the time of data collection so we cannot rule out reverse causality–unemployment caused by disability. The study considered G2D as visible disability detected at the clinical examination performed by the study team, did not distinguish physical disabilities detected during treatment or after MDT completion, however, accurately describes the visible deformities in the group assessed, evidencing the invisible nature of a concerning condition in a population vulnerable to developing further secondary disabilities, functional decline with consequent repercussions on their lives. Therefore, despite the limitations, the findings indicate a significant challenge to health services that should focus more on: planning leprosy surveillance, monitoring disabilities and providing continued leprosy care to patients who concluded MDT aiming to reduce the burden of disabilities among leprosy patients.

### Conclusions

This study shows that physical disabilities remain after completion of MDT and frequently occur in an endemic area in Brazil. Finding new ways to reduce the burden of disability are urgently needed, and may include systematic follow-up of patients after treatment completion combined with evidence-based preventative measures.

## Supporting information

S1 Appendix Assessment of disability and nerve function. (PDF)

S1 Checklist STROBE Checklist. (PDF)
