# Supplementary material for: Grade 2 disabilities in leprosy patients from Brazil: Need for follow-up after completion of multidrug therapy
Source: PLoS Negl Trop Dis. 2018 Jul 16;12(7):e0006645. doi: 10.1371/journal.pntd.0006645 (PMC6062121; doi:10.1371/journal.pntd.0006645)
Supplement: S1 Appendix — (PDF) [file pntd.0006645.s001.pdf]

S1 Appendix. Assessment of disability and nerve function.

IntegraHans	
Patient (ID): ______________________	SINAN number
Examiner: ____	Collection date: ______________
Revisor: ____	Review date: ________________

ITEM	VARIABLE	CODE		Revisor
1.	Patient name	____		
2.	Date of birth	_____ / _____ / ________		
3.	Gender	Male<br>Female	1<br>2	( )
4.	Current occupation	____		
5.	Operational classification	Paucibacillary<br>Multibacillary<br>Undefined	1<br>2<br>9	( )
6.	Start date of MDT	_____ / _____ / ________		
7.	Completion date of MDT	_____ / _____ / ________		
8.	Clinical forms	Indeterminate<br>Tuberculoid<br>Borderline<br>Lepromatous<br>Undefined	1<br>2<br>3<br>4<br>9	( )

FACE	1 / /		2 / /		3 / /	
**Nose**	**R**	**L**	**R**	**L**	**R**	**L**
Chief complaint						
Dry skin (Y/N)						
Wound (Y/N)						
Septal perforation (Y/N)						
**Eyes**	**R**	**L**	**R**	**L**	**R**	**L**
Chief complaint						
Lagophtalmos mild (Y/N mm)						
Lagophtalmos severe (Y/N mm)						
Trichiasis (Y/N) / Ectropion (Y/N)						
Corneal hypoesthesia (Y/N)						
Corneal opacity (Y/N)						
Cataract (Y/N)						
Poor visual acuity (VA < 6/60)						

Note: Y = Yes; N = No. If lagophthalmos (register in mm). For visual acuity: N/C = no correction; W/C = with correction

UPPER LIMBS	1 / /		2 / /		3 / /	
Chief complaint						
**Nerve palpation**	**R**	**L**	**R**	**L**	**R**	**L**
Ulnar						
Median						
Radial						

Note: N = Normal; TH = Thickened; TD = Tenderness

Voluntary muscle test		1 / /		2 / /		3 / /	
		**R**	**L**	**R**	**L**	**R**	**L**
Ulnar	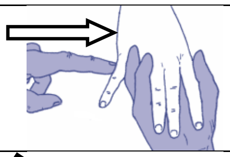						
Median	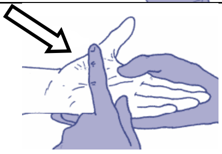						
Radial	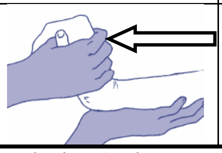						

Caption: Medical Research Concil - Grade 0 - No muscle activation; Grade 1 - Trace muscle activation, such as a twitch, without achieving full range of motion; Grade 2 - Muscle activation with gravity eliminated, achieving full range of motion; Grade 3 - Muscle activation against gravity, full range of motion; Grade 4 - Muscle activation against some resistance, full range of motion; Grade 5 - Muscle activation against examiner's full resistance, full range of motion

Sensory testing					
1 / /		2 / /		3 / /	
R	L	R	L	R	L

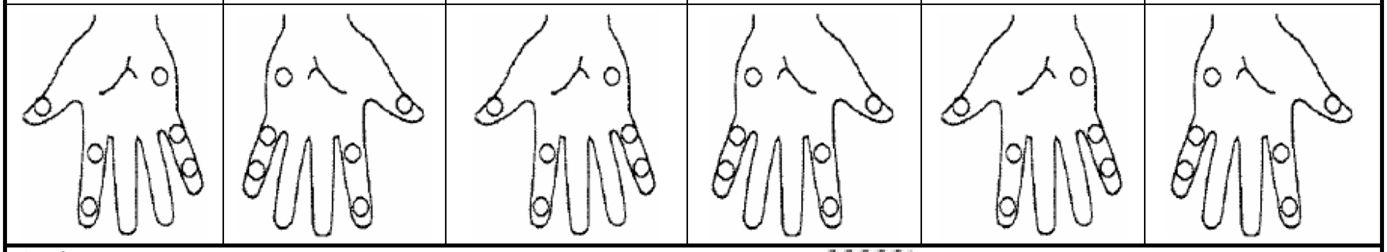

Caption:
Clawing: Mobile = M Rigid = R Wounds/ulcers: 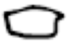 **Reabsorption:** 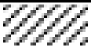 **Monofilaments: Standardized code**

LOWER LIMBS	1 / /		2 / /		3 / /	
Chief complaint						
**Nerve palpation**	**R**	**L**	**R**	**L**	**R**	**L**
Common fibular						
Posterior tibial						

Note: N = Normal; TH = Thickened; TD = Tenderness

Voluntary muscle test		1 / /		2 / /		3 / /	
		**R**	**L**	**R**	**L**	**R**	**L**
Hallucis: Elevation, extension (fibular nerve)	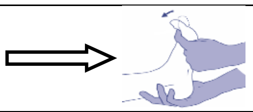						
Foot: Elevation, dorsiflexion. (fibular nerve)	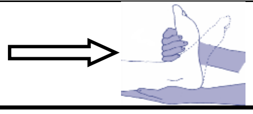						

Caption: Medical Research Concil - Grade 0 - No muscle activation; Grade 1 - Trace muscle activation, such as a twitch, without achieving full range of motion; Grade 2 - Muscle activation with gravity eliminated, achieving full range of motion; Grade 3 - Muscle activation against gravity, full range of motion; Grade 4 - Muscle activation against some resistance, full range of motion; Grade 5 - Muscle activation against examiner's full resistance, full range of motion

Sensory testing					
1 / /		2 / /		3 / /	
R	L	R	L	R	L

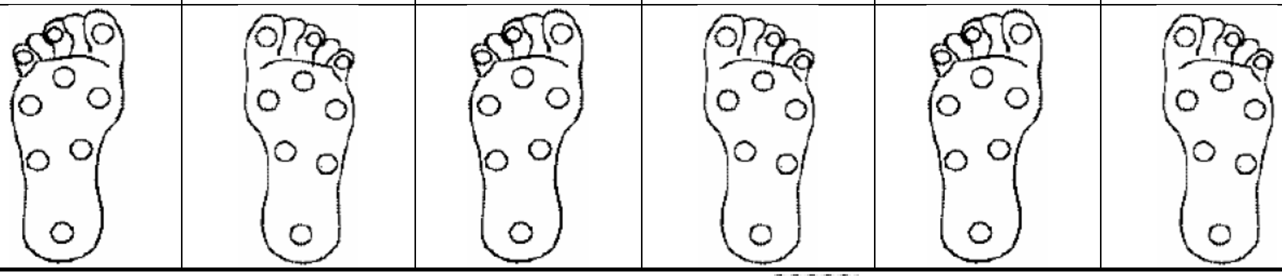

Caption:
Clawing: Mobile = M Rigid = R Wounds/ulcers: 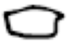 **Reabsorption:** 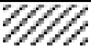 **Monofilaments: Standardized code**

WHO DG	*EYE*							*HAND*							*FOOT*						
	Signs and symptoms	*1*		*2*		*3*		*Signs and symptoms*	*1*		*2*		*3*		*Signs and symptoms*	*1*		*2*		*3*	
		R	*L*	*R*	*L*	*R*	*L*		*R*	*L*	*R*	*L*	*R*	*L*		*R*	*L*	*R*	*L*	*R*	*L*
**0**	**No eye problems due to leprosy; no evidence of visual loss**							**No anaesthesia, no visible deformity or damage**							**No anaesthesia, no visible deformity or damage**						
**1**	**Eye problem due to leprosy present, but vision not severely affected as a result (vision 6/60 or better; can count fingers at 6 metres)**							**Anaesthesia, but no visible deformity or damage**							**Anaesthesia, but no visible deformity or damage**						
**2**	**Lagophtalmos and/or ectropion**							**Loss of tissue, wounds ulcer**							**Loss of tissue, wounds, ulcer**						
	**Trichiasis**							**Claw hand**							**Claw toe**						
	**Corneal opacity**							**Absorption**							**Absorption and/or contracture**						
	**Severe visual impairment (vision worse than 6/60; inability to count fingers at 6 metres), lagophthalmos, iridocyclitits, corneal opacities**							**Wrist drop**							**Foot drop**						

Date	WHO Disability Grade (DG)	Eyes		Hands		Feet		Maximum DG	EHF Score (RE + LE + RH + LH + RF + LF)	Signature
		R	L	R	L	R	L			
1 ____/___/____	Grade									
	EHF Sum									
2 ____/___/____	Grade									
	EHF Sum									
3 ____/___/____	Grade									
	EHF Sum									
